# The effects of intimate relationship characteristics on unprotected anal intercourse among same-sex male couples in China: a dyadic analysis using the actor-partner interdependence model

**DOI:** 10.1186/s12879-021-06317-y

**Published:** 2021-06-22

**Authors:** Sha Chen, Qingling Yang, Juan He, Xiongzhi Fan, Zhongqi Liu, Jialing Qiu, Zhiwei Zheng, Jing Gu, Weibin Cheng, Yuantao Hao, Jinghua Li, Chun Hao

**Affiliations:** 1 Department of Medical Statistics, School of Public Health, Sun Yat-sen University, Guangzhou, 510080 China; 2 Institution of Drug Clinical Trial, Guangdong Second Provincial General Hospital, Guangzhou, 510317 Guangdong China; 3 Sun Yat-sen Global Health Institute, School of Public Health & Institute of State Governance, Sun Yat-sen University, Guangzhou, 510080 China; 4 Health Information Research Center & Guangdong Key Laboratory of Medicine, School of Public Health, Sun Yat-sen University, Guangzhou, 510080 China; 5 Institute for Healthcare Artificial Intelligence Application, Guangdong Second Provincial General Hospital, Guangzhou, 510317 Guangdong China

**Keywords:** Men who have sex with men, Intimate relationship characteristics, Unprotected anal intercourse, Actor-partner interdependence model, Dyadic data

## Abstract

**Background:**

Unprotected anal intercourse (UAI) within the context of concurrent sexual relationship are prevalent among men who have sex with men (MSM) who have regular male sex partners and it aggravates the risk of HIV infection among this community. The aim of this study was to assess the effect of intimate relationship characteristics on UAI among MSM couples at the dyadic level.

**Methods:**

Two hundred four MSM couples were recruited from a HIV testing clinic from April 2017 to April 2018 in Guangzhou, China. The actor-partner interdependence model (APIM) was applied for dyadic analysis. Each MSM couple was divided into the insertive role and the receptive role according to their regular anal sex role. In this context, actor effect is the impact of an MSM’s intimate relationship characteristics on his own UAI, and partner effect is the impact of his partner’s intimate relationship characteristics on his UAI.

**Results:**

Of the 408 participants, 58.82% had UAI with regular male sex partner (UAI-RP) and 8.09% had concurrent UAI. Intimate relationship characteristics were associated with concurrent UAI, but not associated with UAI-RP. For the receptive role, his relationship investment exerted significant actor and partner effects on concurrent UAI (*AOR*_actor_ = 1.31, *P* < 0.001; *AOR*_*partner*_ = 1.17, *P* < 0.001). Meanwhile, receptive role’s violence experience within relationship exerted significant actor effects on his own concurrent UAI (*AOR*_actor_ = 6.43, *P* = 0.044).

**Conclusions:**

Relationship investment and violence experience influenced concurrent UAI among MSM couples and it varied in different sex roles. Additional assistance on empowerment, relationship therapy and sexual agreement is urgently needed to reduce their high possibility on engagement of HIV-related risk behaviors.

**Supplementary Information:**

The online version contains supplementary material available at 10.1186/s12879-021-06317-y.

## Introduction

The HIV epidemic was disproportionally severe among men who have sex with men (MSM) in middle-upper-income countries [1–5]. HIV prevalence rate among MSM increased from 1.4% in 2001 to 7.8% in 2016 in China [6, 7]. A mathematical study revealed that new HIV infections among MSM attributed to regular male sex partner (RP) increased from 34 to 40% during 2002–2010 in China [8]. The high HIV transmissions among MSM with RP were attributed to frequent unprotected anal intercourse (UAI) within trust-based intimate relationships [9–12] and concurrent sexual partnerships [13–15]. Because of trust, studies among MSM showed that the prevalence of UAI with regular partners is relatively higher than that with casual partners globally. For example, the prevalence of UAI with RP versus casual partners was 37.0–87.0% vs. 13.0–25.0% in United States [16–19], 43.0–53.9% vs. 23.6–33.0% in China [20–23], 63.4% vs. 18.7% in UK [24], and 46.3% vs. 30.7% in Australia [25], respectively. What’s more, concurrent sexual partnerships with high-risk sexual behaviors are very common in MSM community. The prevalence of concurrent UAI among MSM was 11–45% [15, 17, 26], 20.7% [23], 30.3% [27], 51% [14] in United States, China, Israel and Vietnam respectively. These concurrent sexual relationships with more than one person increase one’s risk of transmitting HIV from one sexual partner to another.

Based on previous studies, intimate relationship characteristics were strong predictors of UAI among MSM [23, 28]. Trust and intimacy were positively associated with UAI with RP because of the perception of partners’ being honest with them [21]. The open sexual agreement was associated with more outside sexual activity and therefore increase the opportunity of UAI with non-regular male sex partners (NRP) [18]. Relationship satisfaction, commitment and communication were negatively associated with UAI with outside partners [29]. Relationship investment reduced the possibility of UAI within and outside the relationship [30]. However, most of these studies set individual MSM as a unit of analysis.

**Fig. 1:**
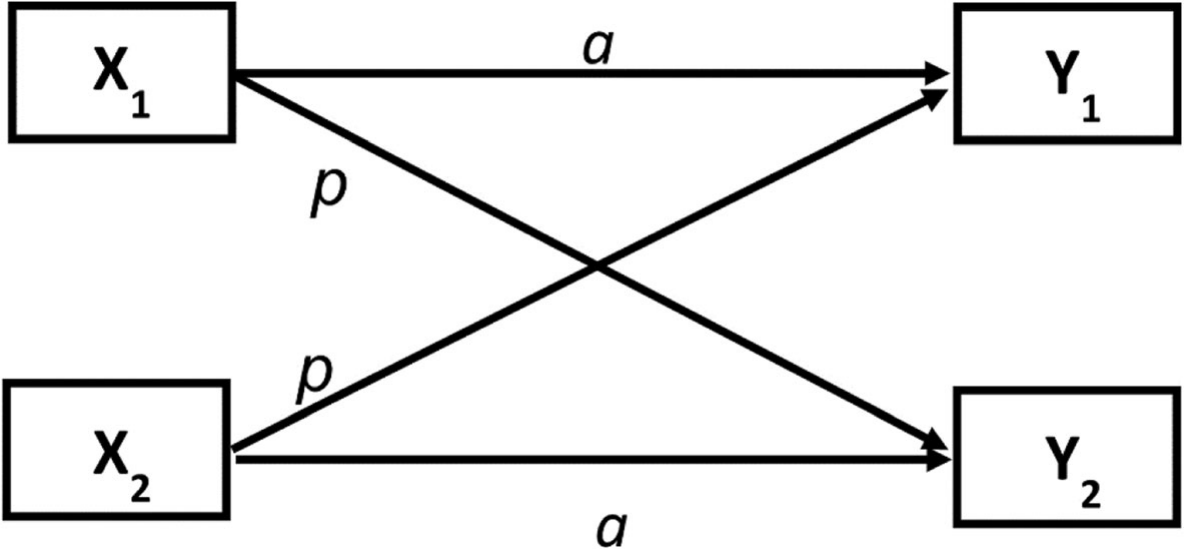
APIM framework. *a* represents actor effects, *p* represents partner effects, X_1_ and Y_1_ are one member’s predictor and outcome scores, and X_2_ and Y_2_ are the other member’s predictor and outcome scores

Few studies have analyzed at the dyadic level, which can address the individual effects of both partners and account for the effect of interdependence within the couple. The actor-partner interdependence model (APIM) is an innovative approach designed to analyze the dyad data and simultaneously estimates the actor and partner effects [31]. The actor effect assesses the degree to which a person’s outcome variable is influenced by his/her own predictors, whereas the partner effect assesses the degree to which a person’s outcome is influenced by his/her partner’s predictors [32]. (See Fig. 1) Currently, there are some studies applied APIM among MSM couples to investigate associated factors of sexual relationship quality, intimate partner violence, and depression. The associated factors included sexual agreements, intimacy development, and relationship satisfaction, etc. [33–36]. However, researches that used APIM to explore the associations between intimate relationships and HIV risk behaviors among MSM couples were rare, and more researches were urgently wanted.

The Interdependence theory (IDT) was a powerful framework to describe the intimate relationship characteristics in a systematic way. IDT analyzed the relations between people in terms of situation structure which can affect the behaviors of dyad [37, 38], which fit well with the APIM approach of this dyad data study. The situation structure involved the dimensions of level of dependence, basis of dependence, covariation of interests and information availability [38, 39]. Through IDT, we were able to more systematically analyze the impact of intimate relationship characteristics on UAI among MSM couples.

Therefore, the objective of this study was to apply the APIM approach to analyze actor effects and partner effects of intimate relationship characteristics on UAI in MSM couples. It was hypothesized that: (1) MSM’s stronger relationship dependence, relationship satisfaction and trust will be linked to more HIS OWN UAI with RP and less concurrent UAI. But stronger relationship control and intimate partner violence will increase HIS OWN UAI with RP and concurrent UAI simultaneously. Better sexual communication and relationship investment will decrease HIS OWN UAI with RP and concurrent UAI (actor effect); (2) MSM’s relationship dependence, relationship satisfaction, trust, relationship control, intimate partner violence, sexual communication, and relationship investment will influence HIS PARTNER’s UAI with RP and concurrent UAI as well (partner effect), but the strength and direction of associations will be different from actor effects.

## Methods

### Participants and recruitment

Participants were recruited from April 2017 to April 2018 in an MSM peer-friendly HIV testing service center in Guangzhou, China. The center is a well-known lesbian, gay, bisexual, and transgender (LGBT) community-based organization (Lingnan Partners Community Support Center) and is cooperating with Guangzhou Center for Disease Control and Prevention. MSM couples were eligible if they met the following criteria: (1) aged 18 years or older, (2) having anal intercourse in the past 3 months, (3) at least one partner considering they are in a relationship; (4) the couple’s relationship length was more than 1 month.

The convenience-sampling method was used in recruiting, including Couple-based and individual-based recruitment. MSM couples and individual MSM who came for HIV tests were invited to our study. After informed consent, couples were asked to fill out the electronic questionnaires in separate private rooms. Participants were informed that their responses on questionnaire would be confidential and would not disclose to anyone, including their partners. And individual participant was required to invite his partner to participate in this study. The questionnaires took about 20 min. If participants had any question about the questionnaires, they could consult the staff on site. After the questionnaires were completed, the staff would check the content of the questionnaires immediately. If there was any missing or dubious answer, the staff would immediately ask the MSM and correct it. Each participant would receive a movie ticket voucher (about 5 USD) as a reward.

Finally, 204 MSM couples were successfully recruited, including 189 couples and 15 individual MSM who successfully invited their partners to participate. Each couple was divided into the insertive role and receptive role according to their commonly anal sex role in the relationship. There were 22 couples unmatched with sex roles, among which 10 couples were that one partner reported as insertive/receptive while the other reported as no specific role. In this case, we adjusted the sex role according to their partners’ sex role. For the left 12 couples, they were divided into insertive or receptive role according to the control power in the relationship, which meant the stronger control power the higher possibility to be insertive role.

### Ethical consideration

The study protocol and consent procedures were approved by the Ethics Committee of Sun Yat-sen University. MSM couples were informed of the study aim and their right to quit at any time. The whole process of the study was assured of confidentiality and anonymity. Written consent was obtained from all participants and could be signed with a nickname.

### Measures

#### Background characteristics

Background information collected in this survey included socio-demographic characteristics (age, marital status, years of residence in Guangzhou, education level, student or not, and monthly income), MSM-related characteristics (sexual orientation, disclosure of the sexual orientation, way to recruit regular partner and substance abuse), relationship background characteristics (relationship length, disclosure of the relationship to their parents, condom use during their first sexual intercourse, relationship type and sexual agreement), HIV testing information (HIV testing experience and partner’s previous HIV status) and sex partnership information (whether had casual male sex partner and multiple regular male sex partners). All these variables were binary.

#### Unprotected anal intercourse information

Participants were asked about the UAI with specific types of sex partners in the past 3 months. A regular partner (RP) was defined as the person the participant had a relationship with, in other words, the boyfriend of the participant. Concurrent UAI was defined as that had UAI with both RP and other male sex partners. The two types of UAI were used as outcome variables.

#### Intimate relationship characteristics

IDT involved the dimensions of level of dependence, the basis of dependence, covariation of interests and information availability. Level of dependence referred to the degree to which an individual relied on his partner and was measured by the Depend subscale of the Adult Attachment Scale (AAS) [40]. Item scores were summed and ranged from 3 to 15 and higher scores indicated a higher level of dependence. Basis of dependence described the way that the couples depend on each other, which included actor control, partner control, and joint control. It was measured by the Relationship Control subscale of the Sexual Relationship Power Scale (SRPS) [41]. The average score of all items was computed for each participant and a higher score indicated higher tendency of actor control. Covariation of interests described whether the interests of the behavioral outcome were equivalent to each other and whether corresponding interest or conflicting of interests. It was evaluated by the scores of relationship satisfaction (higher scores indicated a higher level of relationship satisfaction) and whether they experienced intimate partner violence. Participants who have experienced any of physical violence, verbal violence or sexual violence in the past 3 months were defined as experienced intimate partner violence. Information availability described partners’ communication related to their relevant needs, goals, and motives and was measured by the Dyadic Sexual Communication Scale (DSC) [42]. The sum score of all items was calculated for each partner and higher scores indicated better sexual communication. Furthermore, we evaluated the trust and relationship investment of the couple to the questionnaires based on the literature review [21, 30, 43]. The trust was measured by The Dependability subscale of the Trust Scale [44]. Item scores were summed and higher scores indicated a higher level of trust. The relationship investment was measured by the three selective items in the Investment Size subscale of the Invested Model Scale [45]. Item scores were summed and a higher score indicated more investment in this relationship. The scales according to IDT we selected were based on the results of the previous qualitative study, literature search, pretest reliability and validity evaluation. We found a few studies using scales among postpartum women, students and heterosexual couples in China [46–48], but MSM relevant studies with the scales were applied abroad [49, 50]. (The details of scales’ each item and Cronbach’s were presented in Table 1. Other basic questions are attached in the supplementary file.)

**Table 1:** Measurement scales to assess intimate relationship characteristics

Variables	Measurements
Dependence measurement: Depend subscale of Adult Attachment Scale (range from 3 to 15)	Three selective items in the Depend subscale of the Adult Attachment Scale (AAS) were used according to the understanding of definition and the reliability test (Cronbach’s α = 0.659). Item 1: People are never there when you need them. Item 2: I find it difficult to trust others completely. Item 3: I am not sure that I can always depend on others to be there when I need them. Participants respond to each item on a 5-point Likert scale (1 = Strongly Disagree, 5 = Strongly Agree). The sum score of all items is calculated for each participant and higher score indicate higher level of dependence.
Relationship control measurement: Relationship Control subscale of Sexual Relationship Power Scale (range from 1 to 4)	Relationship Control subscale of the Sexual Relationship Power Scale (SRPS) were used (Cronbach’s α = 0.803). Item 1: If I asked my partner to use a condom, he would get violent. Item 2: If I asked my partner to use a condom, he would get angry. Item 3: Most of the time, we do what my partner wants to do. Item 4: My partner won’t let me wear certain things. Item 5: When my partner and I are together, I’m pretty quiet. Item 6: My partner has more say than I do about important decisions that affect us. Item 7: My partner tells me who I can spend time with. Item 8: If I asked my partner to use a condom, he would think I’m having sex with other people. Item 9: I feel trapped or stuck in our relationship. Item 10: My partner does what he wants, even if I do not want him to. Item 11: I am more committed to our relationship than my partner is. Item 12: When my partner and I disagree, he gets his way most of the time. Item 13: My partner gets more out of our relationship than I do. Item 14: My partner always wants to know where I am. Item 15: My partner might be having sex with someone else. Participants respond to each item on a 4-point Likert scale (1 = Strongly agree, 4 = Strongly Disagree). The average score of all items is calculated for each participant and higher score indicate higher tendency of actor control.
Relationship satisfaction measurement (range from 0 to 10)	Item: What is your self-scoring of your satisfaction with your intimate relationship? The full score is 10 and higher score indicate higher level of relationship satisfaction.
Sexual communication measurement: dyadic Sexual Communication Scale (range from 8 to 48)	The Dyadic Sexual Communication Scale (DSC) was used (Cronbach’s α =0.853). Item 1: My partner rarely responds when I talk about our sex life. Item 2: Some sexual matters are too upsetting to discuss with my sexual partner. Item 3: There are sexual issues or problems in our sexual relationship that we have never discussed. Item 4: My partner and I never seem to resolve our disagreements about sexual matters. Item 5: Whenever my partner and I talk about sex, I feel like she or he is lecturing me. Item 6: My partner often complains that I am not very clear about what I want sexually. Item 7: My partner and I have never had a heart-to-heart talk about what I want sexually. Item 8: Even when angry with, my partner is able to appreciate my views on sexuality. Participants respond to each item on a 6-point Likert scale (1 = Strongly Agree, 6 = Strongly Disagree). The sum score of all items is calculated for each participant and higher score indicate better communication.
Trust measurement: Dependability subscale of Trust Scale: (range from −15 to 15)	The Dependability subscale of the Trust Scale was used (Cronbach’s α = 0.859). Item 1: My partner has proven to be trustworthy and I am willing to let him/her engage in activities which other partners find too threatening. Item 2: I have found that my partner is unusually dependable, especially when it comes to things which are important to me. Item 3: I am certain that my partner would not cheat on me, even if the opportunity arose and there was no chance that he/she would get caught. Item 4: I can rely on my partner to keep the promises he/she makes to me. Item 5: Even when my partner makes excuses which sound rather unlikely, I am confident that he/she is telling the truth. Participants respond to each item on a 7-point Likert scale (−3 = Strongly Disagree, 3 = Strongly Agree). The sum score of all items is calculated for each participant and higher score indicate higher level of trust.
Relationship investment measurement: investment model Scale (range from 0 to 24)	Three selective items in the Investment Size subscale of the Invested Model Scale (IMS) were used according to the understanding of definition and the reliability test (Cronbach’s α = 0.802). Item 1: I have put a great deal into our relationship that I would lose if the relationship were to end. Item 2: Many aspects of my life have become linked to my partner (recreational activities, etc.), and I would lose all of this if we were to break up. Item 3: Compared to other people I know, I have invested a great deal in my relationship with my partner. Participants respond to each item on a 9-point Likert scale (0 = Strongly Disagree, 8 = Strongly Agree). The sum score of all items is calculated for each participant and higher score indicate more investment in this relationship.

### Data analysis

Paired-sample *t* test and McNemar’s *χ*^2^ test were used to detect the differences between insertive role and receptive role.

The APIM with distinguishable dyads [51] was applied to examine the influence of insertive role and receptive role’s intimate relationship characteristics on UAI. General estimate equation (GEE) was used for estimating the APIM. Two basic equations are as follows:

$$ \mathrm{logit}{\left(\Pr \left(\mathrm{Y}=1\right)\right)}_{Insertive}={\upbeta}_{0\  Insertive}+{a}_{Insertive}\ast {X}_{Insertive}+{p}_{Insertive- Receptive}\ast {X}_{Receptive} $$

$$ \mathrm{logit}{\left(\Pr \left(\mathrm{Y}=1\right)\right)}_{Receptive}={\upbeta}_{0\  Receptive}+{a}_{Receptive}\ast {X}_{Receptive}+{p}_{Receptive- Insertive}\ast {X}_{Insertive} $$

In the two equations above, Y represents the UAI with specific types of sex partners and it’s a binary variable. In the first equation, logit(Pr(Y = 1))_*Insertive*_ is the insertive role’s logit of the probability of an outcome occurred; *X*_*Insertive*_ and *X*_*Receptive*_ are intimate relationship characteristics of the insertive role and receptive role; *a*_*Insertive*_ is the effect of insertive role’s intimate relationship characteristics on their own outcome variables (actor effect), and *p*_*Insertive* − *Receptive*_ is the effect of the receptive role’s intimate relationship characteristics on insertive role’s outcome variables (partner effect); β_0 *Insertive*_ is the intercept of the first equation. The second equation can be interpreted in the same way.

First, univariate odds ratios (*OR*) and 95% confidence intervals (95% *CI*) of all actor and partner effects were used to describe the associations between independent variables and outcome variables. At the second step, background characteristics with a *P-value* smaller than 0.10 in univariate APIM were adjusted. And adjusted OR (*AOR*) and 95% *CI* were derived from multivariate APIM of intimate relationship characteristics variables. *P* < 0.05 was considered statistically significant. Statistical analysis was performed on SAS (SAS 9.4 for Windows; SAS Institute Inc., NC).

## Results

### Background characteristics

The majority of the 408 participants were above 25 years, unmarried, lived in Guangzhou for more than 1 year, had post-secondary or higher education levels, earned more than 5000 RMB (about 750 USD) per month and were not students. Regarding to sexual orientation, 82.84% were homosexual identified, 65.69% disclosed their sexual orientation to persons who were from outside of MSM community. A total of 78.68% found RP via Internet, 23.28% had substance abuse. As regards the MSM couples’ relationship background characteristics, 38.73% couples had maintained relationship for more than 1 year, 13.24% disclosed their relationship to their parents, 76.47% used condoms during their first sexual intercourse, 80.88% couples’ relationship type was monogamous but only 33.33% couples reported having sexual agreement. Of the 408 participants, 87.26% had HIV testing experience, 69.61% reported their partners’ previous HIV status was negative. 19.36% had casual male sex partners and 11.52% had multiple RP.

Compared with receptive role, insertive role was more likely to earn more (62.25% VS. 51.96%, *P* = 0.026) and have HIV testing experience (91.67% VS. 82.84%, *P* = 0.010), whereas was less likely to be a student (13.24% VS. 20.10%, *P* = 0.038), substance abuser (19.61% VS. 26.96%, *P* = 0.032) and homosexual-identified (76.96% VS. 88.73%, *P* = 0.001). Other variables were comparable between the different sex roles (all *P* > 0.05) (Table 2).

**Table 2:** Background characteristics of the 204 MSM couples (*N* = 408)

Variables	Total (***N*** = 408) % (n)	Insertive role (n_**1**_ = 204) % (n)	Receptive role (n_**2**_ = 204) % (n)	***P***-value
***Socio-demographic characteristics***				
Age (years) > 25	64.22 (262)	68.63 (140)	59.80 (122)	0.050^†^
Currently unmarried	91.18 (372)	92.16 (188)	90.20 (184)	0.541
Lived in Guangzhou > 1 year	86.77 (354)	86.76 (177)	86.76 (177)	1.000
Post-secondary education level or above	82.60 (337)	85.29 (174)	79.90 (163)	0.152
Currently a student	16.67 (68)	13.24 (27)	20.10 (41)	0.038^*^
Monthly income >5000RMB (about 750 USD)	57.11 (233)	62.25 (127)	51.96 (106)	0.026^*^
***MSM-related characteristics***				
Self-identified as homosexual orientation	82.84 (338)	76.96 (157)	88.73 (181)	0.001^**^
Disclosed the sexual orientation to persons who were from outside of MSM community	65.69 (268)	66.67 (136)	64.71 (132)	0.731
Recruit regular male sex partners via Internet	78.68 (321)	78.43 (160)	78.92 (161)	1.000
Substance abuse	23.28 (95)	19.61 (40)	26.96 (55)	0.032^*^
***Relationship background characteristics***				
Relationship length > 1 year	38.73 (158)	38.73 (79)	38.73 (79)	–
Disclosed the relationship to their parents	13.24 (54)	13.24 (27)	13.24 (27)	–
Used condoms during their first sexual intercourse	76.47 (312)	76.47 (156)	76.47 (156)	–
Monogamous sexual relationship	80.88 (330)	80.88 (165)	80.88 (165)	–
Had sexual agreement	33.33 (136)	33.33 (68)	33.33 (68)	–
***HIV testing information***				
Had ever tested HIV before	87.26 (356)	91.67 (187)	82.84 (169)	0.010^**^
Partner’s previous HIV status				0.894
Negative	69.61 (284)	70.10 (143)	69.12 (141)	
Positive or unknown	30.39 (124)	29.90 (61)	30.88 (63)	
***Sex partnership information***				
Had casual male sex partner (s)	19.36 (79)	20.10 (41)	18.63 (38)	0.780
Had multiple regular male sex partners	11.52 (47)	11.76 (24)	11.27 (23)	1.000

^†^*P* < 0.10; ^*^P < 0.05; ^**^P < 0.01; Statistically significant results were bolded

### Unprotected anal intercourse information

The reported UAI-RP was unanimous within 168 couples, with 84 couples both reported had UAI-RP, and 84 couples both reported not had UAI-RP. However, 36 couples gave inconsistent reports to UAI-RP. In this case, one partner reported had UAI-RP, we classified the couple as having UAI-RP. Finally, out of 204 couples, 120 couples had UAI-RP and the prevalence rate was 58.82%. The prevalence of concurrent UAI was 8.09%. All types of UAI were comparable between insertive role and receptive role (all *P* > 0.05) (Table 3).

**Table 3:** UAI information and intimate relationship characteristics of the 204 MSM couples (*N* = 408)

Variables	Total (N = 408) Mean (SD) or % (n)	Insertive role (n_**1**_ = 204) Mean (SD) or % (n)	Receptive role (n_**2**_ = 204) Mean (SD) or % (n)	***P***-value
***UAI information***				
Had UAI with regular partner	58.82 (240)	58.82 (120)	58.82 (120)	–
Had concurrent UAI	8.09 (33)	8.33 (17)	7.84 (16)	1.000
***Intimate relationship characteristics***				
Dependence	8.83 (2.40)	8.92 (2.57)	8.74 (2.21)	0.472
Relationship control	2.84 (0.38)	2.83 (0.37)	2.84 (0.39)	0.824
Relationship satisfaction	8.47 (1.59)	8.45 (1.65)	8.49 (1.54)	0.763
Sexual communication	35.54 (6.79)	35.61 (6.91)	35.48 (6.69)	0.811
Trust	6.27 (5.34)	6.28 (5.54)	6.26 (5.15)	0.975
Relationship investment	13.48 (5.77)	13.30 (5.90)	13.65 (5.63)	0.518
Intimate partner violence	23.28 (95)	25.49 (52)	21.08 (43)	0.233

### Intimate relationship characteristics

Out of the 408 MSM, the mean scores of the selected five measurement scales of intimate relationship characteristics (dependence, relationship control, sexual communication, trust, relationship investment) were 8.83, 2.84, 35.54, 6.27, and 13.48, respectively. In addition, the mean score of the items that reflected the relationship satisfaction was 8.47. And 23.28% (95 out of 408) MSM experienced intimate partner violence in the past 3 months. Specifically, out of 408 MSM, 2.94% experienced physical violence, 22.30% experienced verbal violence and 1.23% experienced sexual violence. All the intimate relationship characteristics were comparable between insertive role and receptive role (all *P* > 0.05) (Table 3).

### Intimate relationship characteristics associated with UAI with RP

In the APIM analysis, the prevalence of UAI-RP among couple participants of the insertive role and receptive role was the same, so the insertive role’s actor/partner effects were equivalent with receptive role’s partner/actor effects. As regards intimate relationship characteristics, the univariate APIM detected several actor and partner effects. For receptive role, his relationship control (*OR*_*actor-Receptive*_ = *OR*_*partner-Insertive*_ = 0.38, 95% *CI*: 0.16–0.92, *P* = 0.031) and violence experience (*OR*_*actor-Receptive*_ = *OR*_*partner-Insertive*_ = 2.76, 95% *CI*: 1.19–6.41, *P* = 0.018) exerted significant actor and partner effects on UAI-RP. After adjusting the significant background variables, no intimate relationship characteristics variables remained significant for both insertive and receptive roles. (Table 4).

**Table 4:** Intimate relationship characteristics associated with UAI of the 204 MSM couples (*N* = 408)

Variables	UAI with regular partner^**a**^				Concurrent UAI^**b**^							
	Insertive role				Insertive role				Receptive role			
	Actor effect		Partner effect		Actor effect		Partner effect		Actor effect		Partner effect	
	***OR*** (95%***CI***)	***AOR*** (95%***CI***)	***OR*** (95%***CI***)	***AOR*** (95%***CI***)	***OR*** (95%***CI***)	***AOR*** (95%***CI***)	***OR*** (95%***CI***)	***AOR*** (95%***CI***)	***OR*** (95%***CI***)	***AOR*** (95%***CI***)	***OR*** (95%***CI***)	***AOR*** (95%***CI***)
Dependence	1.02 (0.91,1.10)	1.00 (0.89,1.12)	0.98 (0.86,1.11)	0.96 (0.84,1.10)	0.88 (0.72,1.08)	0.84 (0.68,1.05)	1.10 (0.86,1.40)	0.96 (0.81,1.14)	1.21 (0.89,1.66)	1.30 (0.83,2.04)	0.92 (0.76,1.11)	0.91 (0.74,1.11)
Relationship control	0.92 (0.41,2.08)	0.81 (0.37,1.78)	0.38* **(0.16,0.92)**	0.51 (0.21,1.24)	0.50 (0.13,1.96)	0.50 (0.08,3.08)	0.43 (0.08,2.22)	0.64 (0.24,1.70)	0.58 (0.10,3.19)	0.46 (0.12,1.77)	1.05 (0.28,3.89)	1.56 (0.27,8.82)
Relationship satisfaction	1.12 (0.93,1.35)	1.08 (0.90,1.29)	0.84 (0.68,1.05)	0.84 (0.67,1.05)	0.88 (0.70,1.11)	0.85 (0.64,1.11)	0.88 (0.65,1.20)	0.84 (0.58,1.20)	0.95 (0.61,1.48)	1.15 (0.74,1.78)	0.91 (0.70,1.19)	0.94 (0.61,1.44)
Sexual communication	1.02 (0.98,1.07)	1.01 (0.97,1.06)	1.00 (0.96,1.05)	1.01 (0.96,1.06)	0.94 (0.87,1.01)	0.95 (0.87,1.03)	1.01 (0.93,1.11)	1.01 (0.93,1.10)	1.04 (0.95,1.13)	1.07 (0.97,1.18)	0.94 (0.88,1.01)	1.03 (0.92,1.15)
Trust	1.02 (0.97,1.08)	1.03 (0.97,1.09)	0.98 (0.93,1.04)	0.98 (0.92,1.04)	1.08 (0.97,1.20)	1.09 (0.96,1.23)	0.95 (0.86,1.05)	0.97 (0.86,1.09)	1.03 (0.89,1.18)	1.11 (0.94,1.30)	0.95 (0.87,1.04)	0.95 (0.85,1.07)
Relationship investment	1.02 (0.97,1.07)	1.00 (0.95,1.05)	1.03 (0.98,1.09)	1.02 (0.97,1.07)	0.98 (0.90,1.07)	0.94 (0.85,1.04)	1.11* **(1.00,1.24)**	1.17** **(1.06,1.29)**	1.04 (0.95,1.14)	1.31** **(1.16,1.48)**	1.04 (0.95,1.13)	1.04 (0.90,1.19)
Intimate partner violence	0.73 (0.36,1.50)	0.76 (0.36,1.61)	2.76* **(1.19,6.41)**	2.30† (0.92,5.73)	0.68 (0.21,2.24)	0.38 (0.11,1.38)	1.95 (0.62,6.07)	4.30† (0.90,20.50)	2.01 (0.54,7.43)	6.43* **(1.05,39.26)**	0.30 (0.05,1.62)	0.34 (0.05,2.40)

The prevalence of UAI-RP among couple participants of insertive role and receptive role were the same, so the insertive role’s actor/partner effects were equivalent with receptive role’s partner/actor effects

*AOR:* adjusted odds ratio, adjusting for background variables which were significant or marginally significant in association with UAI:

^a^ Only UAI with regular partner: monthly income, relationship length, used condoms during their first sexual intercourse, had sexual agreement;

^b^ Concurrent UAI: currently a student, sexual orientation, substance abuse, used condoms during their first sexual intercourse, type of the relationship, had sexual agreement, had multiple regular partners

^†^*P* < 0.10; ^*^P < 0.05; ^**^P < 0.01; Statistically significant results were bolded

### Intimate relationship characteristics associated with concurrent UAI

As regards intimate relationship characteristics, the univariate model showed only a significant partner effect whereas no significant actor effects were found. For receptive role, his relationship investment exerted a partner effect on concurrent UAI (*OR*_*partner*_ = 1.11, 95% *CI*: 1.00–1.24, *P* = 0.046). For insertive role, no actor or partner effects were found (Table 4).

**Fig. 2:**
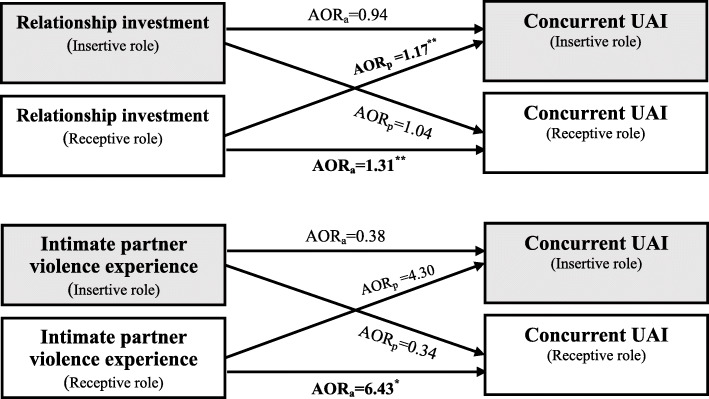
Actor-partner interdependence model of intimate relationship characteristics predicting concurrent UAI in MSM couples (only those with significant AOR were denoted on the Fig. 1). AOR_a_: adjusted odds ratio denoted actor effect of a MSM’s intimate relationship characteristics on his own concurrent UAI; AOR_p_: adjusted odds ratio denoted partner effect of a MSM’s partner’s intimate relationship characteristics on his concurrent UAI. ^†^*P* < 0.10; ^*^P < 0.05; ^**^P < 0.01

After adjusting the confounding variables, the model detected several actor effects and partner effects. For receptive role, his relationship investment (*AOR*_*actor*_ = 1.31, 95% *CI*: 1.16–1.48, *P* < 0.001) and intimate partner violence experience (*AOR*_*actor*_ = 6.43, 95% *CI*: 1.05,39.26, *P* = 0.044) had actor effects on his own concurrent UAI, and his relationship investment also exerted the effect on partner’s concurrent UAI (*AOR*_*partner*_ = 1.17, 95% *CI*: 1.06–1.29, *P* = 0.002). For insertive role, no actor or partner effects were detected (Table 4). Figure 2 showed the results in the form of APIM framework.

## Discussion

This is the first study that applied the APIM to investigate the actor and partner effects of intimate relationship characteristics on UAI in a sample of MSM couples in China. The findings of this study included: (1) intimate relationship characteristics had a certain impact on concurrent UAI at dyadic level, but not associated with UAI-RP; and (2) Intimate relationship characteristics exerted different actor or partner effects on concurrent UAI by different sex roles. For the receptive MSM, those who invested more and experienced violence in the relationship were more likely to have concurrent UAI. The interesting finding was that receptive MSM’s more investment in the relationship also associated with his partner’s high possibility to have concurrent UAI.

The occurrence of UAI-RP (58.8%) was higher than that of concurrent UAI (8.1%). Partnered MSM might consider their relationships to be monogamous and not perceive the risk of HIV infection through UAI-RP [21, 28, 52]. In addition, some MSM couples imagined that UAI could promote their intimacy and relationship quality [52, 53]. Meanwhile, the prevalence of concurrent UAI was lower than that of other researches [15, 26, 54]. One possible reason was that most MSM couples in our study have been in a relationship for a relatively short time about less than 1 year (61.3%), the attractive freshness of RP might prevent the MSM from seeking outside partners.

Inconsistent with some previous researches [18, 21, 29, 55], after adjusting confounders, our results revealed that all actor and partner effects of intimate relationship characteristics on UAI-RP were not significant. Relationship control and intimate partner violence experience were statistically significant in the univariate analysis but were not significant in the multivariate analysis. Our subsequent analysis found that correcting relationship length could change the statistical conclusion (from statistically significant to not statistically significant) in the association between relationship control and UAI-RP. Additionally, correcting the variables of condom use during their first sexual intercourse and the sexual agreement could change the statistical conclusion (from statistically significant to not statistically significant) as well. Therefore, relationship length, whether used condoms during their first sexual intercourse and sexual agreement might be influence factors of UAI-RP.

As regards the outcomes of concurrent UAI, for the receptive role, the increase of relationship investment could increase not only his own but also his partner’s likelihood of engaging in concurrent UAI. A meta-analysis indicated that higher relationship investment was associated with higher relationship commitment. And it was associated with less likelihood of relationship breakup [56]. A high level of investment of the receptive role might let them fatigue in intimate relationships, but they continued to persevere with their relationships. In this case, emotional fatigue might make the receptive role unable to resist the temptation of UAI-NRP. Besides, receptive role’s high investment to an intimate relationship might make insertive role felt that their relationship was stable enough, and then insertive role was more daring to seek sexual stimulation outside and had concurrent UAI. Accordingly, a similar relationship was observed in a sample of MSM couples, who reported that high relationship investment was associated with more UAI in steady MSM relationships [57]. In our study, with the increase of the relationship investment, the receptive role might make a compromise without using condoms to allow his partner to enjoy more sexual pleasure, thus the UAI-RP increased. Therefore, for insertive role and receptive role, the UAI-RP and UAI-NRP both increased, thus the likelihood of concurrent UAI increased.

Our findings also suggested that receptive role’s violence experience within the relationship could increase their own occurrence of concurrent UAI. Limited capacity to negotiate condom use and forced sex [58] might be the mechanism that receptive role engaged with more UAI-RP. Besides, violence experience might drive the receptive role to seek comfort from NRP and therefore engage in UAI-NRP. Therefore, the likelihood of concurrent UAI increased.

In all, the study had potentially important implications for behavioral HIV prevention interventions. First, we should take a dyadic perspective on MSM couples to conduct interventions that target a reduction of UAI among MSM population. Furthermore, HIV intervention should focus on educating adolescent MSM about condom use and promoting healthy sexual agreements. The legal system should be prepared to support homosexual victims of intimate partner violence. MSM should be educated on the harm from intimate partner violence, especially focused on the receptive role.

Our research had several limitations that should be considered when interpreting the findings. First, the cross-sectional nature of the study design limited inferences about the causal relationships between intimate relationship characteristics and UAI. Findings reported here needed to be confirmed in longitudinal studies. Second, the study was conducted only in one HIV testing service clinic in Guangzhou and therefore may affect the generalization. Third, measures were self-reported and might exist some self-reported bias of recall bias.

## Conclusion

This study did a meaningful exploration of the actor and partner effects of intimate relationship characteristics on UAI among MSM couples. The findings analyzed both actor and partner effects of intimate relationship characteristics on UAI, supporting the idea that an individual’s characteristics of intimate relationship could impact not only his own but also his partner’s UAI. We need to pay attention to MSM who are in the relationship and focus on the specific sex role partners during HIV routine work or programs (HIV testing services counseling, online help services, etc.), considering to provide additional assistance to them on empowerment, relationship therapy and sexual agreement to reduce their high possibility on the engagement of HIV-related risk behaviors within or outside the couple relationships.

## Supplementary Information

**Additional file 1.** 2017–2018 Guangzhou MSM questionnaire.

## Data Availability

The datasets generated and analysed during the current study are not publicly available due to the privacy of the participants but are available from the corresponding author on reasonable request.

## Abbreviations

UAI: Unprotected anal intercourse
UAI-RP: Unprotected anal intercourse with regular male sex partner
NRP: Non-regular male sex partners
HIV: Human Immunodeficiency Virus
MSM: Men who have sex with men
APIM: Actor-partner interdependence model
