# Supplementary material for: The effects of intimate relationship characteristics on unprotected anal intercourse among same-sex male couples in China: a dyadic analysis using the actor-partner interdependence model
Source: BMC Infect Dis. 2021 Jun 22;21:593. doi: 10.1186/s12879-021-06317-y (PMC8218385; doi:10.1186/s12879-021-06317-y)
Supplement: Supplementary file 1 — Additional file 1. 2017–2018 Guangzhou MSM questionnaire. [file 12879_2021_6317_MOESM1_ESM.docx]

Subscribe to DeepL Pro to edit this document.Visit www.DeepL.com/Pro for more information.

| \| **2017-2018 Guangzhou MSM questionnaire** \| \| --- \|  \|  \| \| --- \| \| \| **1.** Date of Birth: year month (Under 18 years old to end the questionnaire） \| \| --- \| \| \|  \| \| **2.** Have you and your boyfriend been in a confirmed relationship for more than 3 months? \| \| ○ Yes ○ No \| \|  \| \| **3.** In the past 3 months, have you had anal sex with a male? \| \| ○ Yes ○ No \| \|  \| \| **4.** Are you and your partner coming together today? \| \| \| ○ Yes \| ○ No \|  \| \| --- \| --- \| --- \| \| \|  \| \| **5.** Would you like your partner to come and take the survey? \| \| ○ Yes ○ No \| \|  \| \| **6.** Your partner's phone number is (This phone number is only used for information verification and announcement of the lottery results when your boyfriend comes to the survey. ) \| \| _________________________________ \| \|  \| \|  \| \| **7.** Your phone number is (This phone number is only used for information verification and announcement of the lottery results when your boyfriend comes to the survey.) \| \|  \| \| **8.** Heterosexual marriage status \| \| \| ○ Unmarried \| ○ married \| ○ Cohabitation \| ○ Divorced or widowed \|  \| \| --- \| --- \| --- \| --- \| --- \| \| \|  \| \| **9.** Household Registration \| \| ○ Guangzhou   ○ Other areas in the province  ○ Other provinces \| \|  \| \| **10.** Length of residence in Guangzhou \| \| \|  \| o < 3 months \| o 3 ~ 6 months \| o 7 ~12 months \| o 1 year ~ 2 years \| o More than 2 years \|  \| \| --- \| --- \| --- \| --- \| --- \| --- \| --- \| \| \|  \| \|  \| \| **11.** Your education level \| \| \| o Primary school and below \| ○ Junior High School \| o High school or junior college \| ○College undergraduate \| o Graduate student or above \|  \| \| --- \| --- \| --- \| --- \| --- \| --- \| \| \|  \| \| 12. Are you a current student? \| \| \| ○ Yes \| ○ No \|  \| \| --- \| --- \| --- \| \| \|  \| \| **13.** Your total monthly income is \| \| \| o No income \| o 2000RMB and below \| ○ 2001-5000RMB \| ○ 5001-10,000RMB \| \| --- \| --- \| --- \| --- \| \| ○10,000RMB -20,000RMB \| o 20,000RMB or more \|  \|  \| \| \|  \| \|  \| \| **14.** What kind of sexual orientation do you consider yourself to be? \| \| \| o Homosexuality \| o Heterosexuality \| o Bisexual \| o Not sure \|  \| \| --- \| --- \| --- \| --- \| --- \| \| \|  \| \| **15.** Have you ever disclosed your identity as a gay man to anyone outside the gay community? \| \| \| □ Didn't tell anyone \| □Fully disclosed \| □ Spouse \| □ Parents \| □ Relatives other than parents \| \| --- \| --- \| --- \| --- \| --- \| \| □ Friend \| □ Colleagues \| □ Medical staff \| □ Other \|  \| \| \|  \| \| **16.** To which other people outside of the gay community did you disclose your gay identity? \| \|  \| \| _________________________________ \| \|  \| \| **17.** Where do you usually meet same-sex couples who want to develop a regular relationship? \| \| \| o Non-Internet \| o Internet \|  \| \| --- \| --- \| --- \| \| \|  \| \| **18.** On which internet dating platforms do you meet same-sex couples who want to develop a regular relationship? (Please list commonly used platforms, such as Blued, WeChat, etc.) \| \|  \| \|  \| \| **19.** Have you ever been tested for HIV (except for this test)? \| \| ○ No  ○ Tested, remembered the number of times  ○ Tested, but couldn't remember the number of times \| \|  \| \| \| **20.** When was the last test? years ___ months___ \| \| --- \| \| \|  \| \| **21.** Which medications have you used in the last 3 months? \| \| \| □ Not used \| □ Rush \| □ methamphetamine \| □ Ecstasy \| □ Cannabis \| \| --- \| --- \| --- \| --- \| --- \| \| □ K powder \| □ 0 capsules \| □ Heroin \| □ Viagra \| □ Other \| \|  \|  \|  \|  \|  \| \| \|  \| \| **22.** What are the other drugs? \| \| _________________________________ \| \|  \| \| **23.** Apart from your boyfriend, have you had any other regular male sexual partners in the last 3 months? \| \| ○ No ○ Yes ○ Can't remember \| \|  \| \| **24.** Besides boyfriend, how many male regular sexual partners did you have in the last 3 months? \| \| _________________________________ \| \|  \| \| **25.** How often did you use condoms with other male regular sex partners in the last 3 months? \| \| \| ○Never used \| o Occasional use \| o Often used \| o Use every time \|  \| \| --- \| --- \| --- \| --- \| --- \| \| \|  \| \| **26.** Have you had a male casual sex partner in the last 3 months? \| \| \| ○ No \| ○ Yes \|  \| \| --- \| --- \| --- \| \| \|  \| \| **27.** How often did you use condoms with your male casual sex partner in the last 3 months? \| \| \| ○Never used \| o Occasional used \| o Often used \| o Used every time \|  \| \| --- \| --- \| --- \| --- \| --- \| \| \|  \| \| \| **28.** When did you and your boyfriend establish a relationship? year ____ month ____. \| \| --- \| \| \|  \| \| **29.** Have you disclosed your relationship with your boyfriend to your parents? \| \| ○ Neither  ○ I have made it public, but he has not  ○ He has made it public, but I have not  ○ Both have been made public \| \|  \| \|  \| \| **30.** Who is in charge in the relationship, you or your boyfriend? \| \| \| ○ Me \| ○ He \| ○ The same \|  \| \| --- \| --- \| --- \| --- \| \| \|  \| \| **31.** In your sexual relationship, which sexrole you play more? \| \| \| ○ 1 \| ○ 0 \| ○None of them \|  \| \| --- \| --- \| --- \| --- \| \| \|  \| \|  \| \| **32.** Did you use a condom the first time you had sex? \| \| \| ○ Yes \| ○ No \|  \| \| --- \| --- \| --- \| \| \|  \| \| **33.** Have you had sex with your boyfriend in the last 3 months? \| \| \| ○ Yes \| ○ No \|  \| \| --- \| --- \| --- \| \| \|  \| \| **34.** How often have you used condoms in the last 3 months? \| \| \| ○Never used \| o Occasional use \| o Often used \| o Use every time \|  \| \| --- \| --- \| --- \| --- \| --- \| \| \|  \| \| **35.** Your boyfriend’s HIV status is (except for this test) \| \| \| ○ Positive \| ○Negative \| ○I don't know \|  \| \| --- \| --- \| --- \| --- \| \| \|  \| \| **36.** Assuming that this test is positive for your HIV infection, will you inform your partner? \| \| \| ○ Yes \| ○ No \|  \| \| --- \| --- \| --- \| \| \|  \| \|  \| \| **37.** In which of the following ways would you prefer to inform him \| \| ○ In person  ○ Informed by WeChat, QQ or email  ○ Informed by Lingnan partners  ○ Informed by other partners in the circle  ○ Informed by other means \| \|  \| \|  \| \| **38.** Assuming he tested positive for HIV this time and told you, would you end the relationship? \| \| \| o Definitely \| o May be \| o Not sure \| o Probably not \| o Definitely not \|  \| \| --- \| --- \| --- \| --- \| --- \| --- \| \| \|  \| \|  \| \| **39.** Which of the following options best describes your current relationship with your boyfriend. \| \| ○ We only have sex with each other  ○ We can both have sex with other people, but never let each other know  ○ We can both have sex with other people, and we can also have threesome or group, there are no conditions \| \|  \| \| **40.** Do you have a sexual contract with your boyfriend? (Sexual contract: For example, a verbal agreement on what can be done when having sex with each other, or whether you can have sex outside of the relationship, and what you need to do when having sex outside of the relationship, such as whether you need to use a condom, etc.) \| \| ○ No sexual contract  ○ Not explicitly discussed or discussed in detail, implicitly stated or tacitly, more speculative;  ○ Explicitly discussed and discussed in detail \| \|  \| |
| --- | --- | --- | --- | --- | --- | --- | --- | --- | --- | --- | --- | --- | --- | --- | --- | --- | --- | --- | --- | --- | --- | --- | --- | --- | --- | --- | --- | --- | --- | --- | --- | --- | --- | --- | --- | --- | --- | --- | --- | --- | --- | --- | --- | --- | --- | --- | --- | --- | --- | --- | --- | --- | --- | --- | --- | --- | --- | --- | --- | --- | --- | --- | --- | --- | --- | --- | --- | --- | --- | --- | --- | --- | --- | --- | --- | --- | --- | --- | --- | --- | --- | --- | --- | --- | --- | --- | --- | --- | --- | --- | --- | --- | --- | --- | --- | --- | --- | --- | --- | --- | --- | --- | --- | --- | --- | --- | --- | --- | --- | --- | --- | --- | --- | --- | --- | --- | --- | --- | --- | --- | --- | --- | --- | --- | --- | --- | --- | --- | --- | --- | --- | --- | --- | --- | --- | --- | --- | --- | --- | --- | --- | --- | --- | --- | --- | --- | --- | --- | --- | --- | --- | --- | --- | --- | --- | --- | --- | --- | --- | --- | --- | --- | --- | --- | --- | --- | --- | --- | --- | --- | --- | --- | --- | --- | --- | --- | --- | --- | --- | --- | --- | --- | --- | --- | --- | --- | --- | --- | --- | --- | --- | --- | --- | --- | --- | --- | --- | --- | --- | --- | --- | --- | --- | --- | --- | --- | --- | --- | --- | --- | --- | --- | --- | --- | --- | --- | --- | --- | --- | --- | --- | --- | --- | --- | --- | --- | --- | --- | --- | --- | --- | --- | --- | --- | --- | --- | --- | --- | --- | --- |
